# Supplementary material for: Chromothripsis during telomere crisis is independent of NHEJ, and consistent with a replicative origin
Source: Genome Res. 2019 May;29(5):737–49. doi: 10.1101/gr.240705.118 (PMC6499312; doi:10.1101/gr.240705.118)
Supplement: Supplemental Material [file supp_gr.240705.118_Supplemental_file_1.zip › contigs/annotated_contigs/DB47/contig.2.DB47_length_377_mean_cov_4.91777188329.docx]

**DB47_length_377_mean_cov_4.91777188329**

```
GCTTACTTTACAAGAGGCCATAGCACAGTGAGGAAAGATACCTGGCCTGTTTGGGCAAGTCCCTTCCTGGCTTTGAGTCTCTGATTTTG
 >chr9:125126844-125127019 + E=6e-94
TCACCTGAGTGGGGCTGGATTGGGCCTTCTCCTAGGGAGCAGCTGGCTCTGACCCACTGTGGTCTTGCTATTCCCAGACCTTATCT|GG

GATGATGGGAGCTATTGAATGTTCTAAGCAGAATTAGGAGAGTGTTCCACATGCCAGACGAGGGTTGTGGAAACACTTCCTCTTCCTTC
>chr9:125128259-125128461 + E=3e-110
AACTATGGTGGAGAAGGAAGTGGGGTGAGAGGACTTGTATTTTACAGCCCCATAAACTTCCTGAGCAACGTCCCCTAGCCAGAGAATGG

CTCTTTCCCCTGGTTTGTCCGT
```
